# Effects of Environmental Temperature During Cultivation of Broccoli Microgreens on Hematological Parameters, Molecular Markers, and Myocardial Antioxidant Status in Swiss Mice

**DOI:** 10.1002/fsn3.72212

**Published:** 2026-08-24

**Authors:** Vedran Balta, Nikola Ferara, Domagoj Đikić, Dyana Odeh, Anđelo Beletić, Irena Landeka Jurčević, Ivana Šola

**Affiliations:** ^1^ Faculty of Science University of Zagreb Zagreb Croatia; ^2^ Department of Dermatology and Venereology Sestre Milosrdnice University Hospital Centre Zagreb Croatia; ^3^ Laboratory of Proteomics, Internal Diseases Clinic, Faculty of Veterinary Medicine University of Zagreb Zagreb Croatia; ^4^ Faculty of Food Technology and Biotechnology University of Zagreb Zagreb Croatia

**Keywords:** broccoli sprouts, glucosinolate, *hematological parameters*, myocard, polyphenols

## Abstract

Modern lifestyle and diet, characterized by low fruit and vegetable intake, contribute to the rising prevalence of diet‐related disorders, particularly cardiovascular diseases. This highlights the importance of nutrition‐based strategies for maintaining human health. Broccoli microgreens, due to their short growth cycle and high content of bioactive compounds, such as polyphenols and glucosinolates, represent a promising functional food. Although in vivo and in vitro studies have demonstrated beneficial physiological effects of Brassica‐derived compounds, excessive intake may also produce adverse, anti‐nutritional effects. Given that broccoli extracts are frequently consumed in the form of various dietary supplements and commercially available cruciferous vegetables, we investigated the in vivo effects of water extracts from broccoli sprouts (BWE) grown at 23°C and 38°C on hematological parameters and myocardial tissue in healthy Swiss mice. Metabolic, structural, inflammatory, and redox changes were evaluated to determine whether growth temperature alters phytochemical composition and consequently affects blood and myocardial physiology. Administration of BWE at a dose of 300 mg/kg body weight significantly reduced mean platelet volume (*p* ≤ 0.05) and enhanced myocardial antioxidant capacity (*p* ≤ 0.05; *p* ≤ 0.0001). Differences in glucosinolate and polyphenol profiles between treatments were associated with increased nitric oxide (NO) production in mice receiving BWE from sprouts grown at 23°C and elevated total glutathione (tGSH) levels in those treated with BWE from sprouts grown at 38°C (*p* ≤ 0.05). These findings suggest that BWE may exert antiplatelet effects and improve myocardial antioxidant status, indicating its potential role in reducing cardiovascular risk.

## Graphical abstract

Temperature‐dependent phytochemical changes modulate cardioprotective responses in healthy mice.

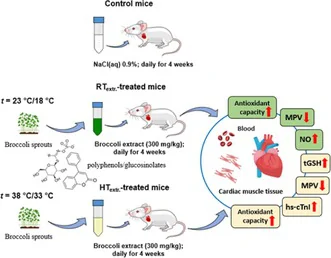

## 1 Introduction

Proper nutrition is essential for maintaining adult health and supporting normal growth and development. Unhealthy lifestyles, characterized by poor dietary habits and insufficient physical activity, are major contributors to morbidity and mortality worldwide. Conditions commonly associated with poor nutrition include type 2 diabetes, dyslipidemia, and cardiovascular diseases such as hypertension, arrhythmias, thrombosis, and stroke. The increasing prevalence of diet‐related disorders has driven interest in functional foods, which, in addition to essential nutrients, provide bioactive compounds such as phytochemicals (Fatima et al. 2024). A growing body of evidence suggests that functional foods can modulate physiological processes and may reduce the risk of chronic diseases (Seth et al. 2025).

Plants from the *Brassicaceae* family (e.g., broccoli, cabbage, kale, and cauliflower) are widely cultivated for human consumption and are valued for their high content of nutrients and bioactive compounds associated with the prevention of chronic diseases. Recent studies indicate that broccoli sprouts (microgreens) may contain 20–50 times higher levels of certain phytonutrients and bioactive compounds compared with mature plants, highlighting their potential as functional foods (Pant et al. 2023). Broccoli sprouts are rich sources of essential nutrients, including vitamins and minerals, as well as bioactive compounds such as polyphenols and glucosinolates (Baenas et al. 2017). These compounds have been associated with antioxidant, anti‐inflammatory, antidiabetic, and anticancer effects. Accordingly, the consumption of microgreens rich in these compounds has been proposed as a dietary strategy for reducing the risk of chronic diseases, including hypercholesterolemia, diabetes, and cardiovascular disorders (Le et al. 2020).

Recent studies in rodent models indicate that broccoli microgreens may improve glucose homeostasis and reduce total serum cholesterol, low‐density lipoprotein (LDL) levels, hepatic expression of inflammatory cytokines, body weight, adipocyte size, progression of atherosclerotic lesions, and hypertension (Kamal et al. 2022; Li et al. 2021; Ma et al. 2022). Evidence from human studies is more limited but suggests similar trends. Consumption of 100 g of broccoli sprouts has been shown to increase high‐density lipoprotein (HDL) levels and reduce total cholesterol compared with baseline values (Connolly et al. 2021). In addition, supplementation with broccoli sprout powder rich in sulforaphane significantly lowered insulin levels in patients with type 2 diabetes (Bahadoran et al. 2012). The effects of Brassica sprouts on blood pressure have also been investigated. In recent years, increasing attention has been directed toward the potential cardiovascular benefits of dietary components, particularly cruciferous vegetables. For example, the addition of 5% (w/w) freeze‐dried cruciferous vegetables to a standard diet has been studied in spontaneously hypertensive and normotensive rats, with both acute (24 h) and chronic (4 weeks) effects on hemodynamic and oxidative‐inflammatory parameters being assessed (Ramirez et al. 2024). Such findings contribute to a better understanding of the role of functional dietary components in the prevention and modulation of hypertension and related inflammatory and oxidative processes.

A critical yet underexplored aspect in this field is the extent to which environmental conditions during plant cultivation influence the biological efficacy of plant‐derived foods in mammalian systems. Environmental factors, particularly temperature, are known to profoundly affect plant primary and secondary metabolism, leading to significant alterations in the biosynthesis and accumulation of phytochemicals (Šola, Gmižić, et al. 2024). Such temperature‐induced metabolic plasticity can substantially modify the nutritional and functional properties of plant‐based foods, ultimately influencing their bioactivity upon consumption. Broccoli sprouts grown under both elevated (38°C/33°C) and reduced (12°C/7°C) temperatures exhibit lower antioxidant capacity and a reduced ability to mitigate H_2_O_2_‐induced oxidative stress in cell models compared to those grown under moderate conditions (23°C/18°C) (Šola, Gmižić, et al. 2024). Higher cultivation temperatures have also been shown to enhance the induction of phase II detoxification enzymes, an effect particularly relevant for mammalian systems. This response has been associated with increased levels of glucosinolates, especially glucoraphanin—a key precursor of sulforaphane, a well‐established inducer of cytoprotective and detoxification enzymes in mammals (Pereira et al. 2002). Although similar temperature‐driven variability in phytochemical composition has been well documented in fruits and other plant systems, studies linking cultivation conditions, phytochemical modulation in microgreens, and subsequent physiological effects in vivo remain scarce. Most in vivo research has focused on post‐harvest processing rather than cultivation conditions. Therefore, despite increasing recognition of environmental regulation of plant metabolomes, it remains unclear how temperature‐driven changes in *Brassica* microgreens translate into physiological responses in mammalian systems (Bricker et al. 2014; Robbins et al. 2010). In particular, the mechanistic links between cultivation temperature, phytochemical remodeling, and systemic effects on hematological and cardiac parameters have yet to be fully elucidated.

In the context of ongoing climate change and the need for resilient, sustainable food systems, understanding how environmental conditions affect the functional quality of rapidly produced crops such as microgreens is of growing importance (Dereje et al. 2023; Le et al. 2020; Turner et al. 2020). Microgreens represent an emerging category of functional foods with high potential for year‐round production in controlled or semi‐controlled environments, yet their biochemical and functional stability under varying growth conditions remains poorly characterized (Pant et al. 2023). Based on this rationale, and given that Brassica microgreens are among the most widely consumed and agriculturally accessible functional food systems, we investigated the in vivo effects of broccoli sprout extracts cultivated under different temperature regimes on hematological parameters and myocardial tissue in healthy Swiss mice. Cultivation was performed at 23°C, corresponding to the average daily environmental temperature in Croatia, and at 38°C, representing peak summer temperature conditions observed in the same geographic region. Building on our previous work demonstrating temperature‐dependent modulation of phytochemical composition in broccoli (Gmižić et al. 2023; Gmižić and Šola 2025; Šola et al. 2023, 2025; Šola, Gmižić, et al. 2024; Šola and Gmižić 2025), we hypothesized that elevated growth temperature would induce substantial alterations in the levels of key bioactive constituents, including polyphenols, glucosinolates, sugars, photosynthetic pigments, and plant hormones. We further hypothesized that such metabolically driven changes would be sufficient to elicit measurable effects on hematological indices, molecular markers, and the antioxidant status of myocardial tissue in consuming animals. Accordingly, this study aimed to elucidate the metabolic, structural, inflammatory, and redox responses induced by temperature‐modulated broccoli sprout extracts, thereby addressing a critical gap in understanding how environmental plant growth conditions influence downstream mammalian physiology.

## 2 Materials and Methods

### 2.1 Plant Growth

We used sprouts grown from broccoli seeds ( *Brassica oleracea* L. convar. *botrytis* (L.) Alef. var. *cymosa* Duch.), purchased from International Seeds Processing GmbH (Quedlinburg, Germany). Prior to germination, the seeds were sterilized using a 2.55% aqueous solution of Izosan for 20 min, after which they were thoroughly washed with autoclaved water to completely remove the disinfectant. Seed germination was carried out in a specialized climate chamber FitoClima 600 PLH (Aralab, Rio de Mouro, Portugal) under strictly controlled conditions of temperature, humidity, and light. Germination was conducted in the dark for 5 days under conditions of 23°C for 16 h and 18°C for 8 h. After germination, the seedlings were divided into two experimental groups: the control group was further cultivated for 7 days under conditions of 23°C (16 h light) and 18°C (8 h dark), while the test group was cultivated for the same duration under conditions of 38°C (16 h light) and 33°C (8 h dark). The humidity was maintained at 65% in all groups. The cultivation was repeated three times to obtain three biological replicates. The aerial parts of the sprouts were collected using sterile scissors, immediately frozen in liquid nitrogen, and dried in a lyophilizer Alpha 1–4 LSCbasic (Martin Christ Gefriertrocknungsanlagen GmbH, Osterode am Harz, Germany). After lyophilization, the plant tissue was ground into powder and stored in a dark, dry, and cold place until chemical analyses.

### 2.2 Preparation and Determination of Quantitative and Qualitative Composition of Extracts

Extracts were prepared from lyophilized plant material in deionized water preheated to 100°C at a concentration of 30 mg/mL. The plant material‐solvent mixture was incubated for 60 min on a digital tube rotator (Thermo Scientific, Shanghai, China) at 20 rpm and room temperature. After incubation, the samples were centrifuged at 11,000 rpm for 10 min using a MIKRO 220 centrifuge (Hettich, Westphalia, Germany). The supernatants were carefully collected and stored at −20°C until further analysis. The material included three biological replicates (two separate groups grown under the same conditions), and three technical replicates were prepared from each biological replicate.

Using appropriate spectrophotometric methods, we determined the concentrations of: (a) total phenolic compounds, (b) flavonoids, (c) flavonols, (d) phenolic acids, (e) hydroxycinnamic acids, and (f) glucosinolates in broccoli extracts using a microplate reader FLUOstar OPTIMA (BMG Labtech, Ortenberg, Germany). Total phenolic compounds were determined using the Folin–Ciocalteu method at a wavelength of 740 nm, as described by Šola, Vujčić Bok, et al. (2024). Total flavonoids were measured at 520 nm according to Nicolescu et al. (2025). Total phenolic acids were analyzed according to Wabaidur (2020) at a wavelength of 485 nm. Total hydroxycinnamic acids and flavonols were analyzed as described by Gmižić et al. (2023) at wavelengths of 320 nm and 355 nm, respectively.

High‐performance liquid chromatography (HPLC) was performed according to Šola et al. (2020) using an Agilent 1100 Series instrument (Agilent, Santa Clara, CA 95051, United States) equipped with a UV/VIS detector to separate, quantify, and identify individual compounds in broccoli sprouts. Prior to analysis, samples were hydrolyzed using 1.2 M HCl at 80°C, 300 rpm for 2 h on a thermoblock Thermo Scientific (Thermo Fisher Scientific Inc., Waltham, Massachusetts, USA). Separation was carried out using a non‐polar Poroshell 120 SB‐C18 column (4.6 × 75 mm, 2.7 μm particle size), and a pre‐column Zorbax Rx‐C18 (4.6 × 12.5 mm, 5 μm particle size). Flavonoids were analyzed at 360 nm, phenolic acids at 310 nm, and *L*‐ascorbic acid at 254 nm. Compound identification was performed by comparing the retention times and spectral profiles of peaks from the samples with those of external standards. Quantification was carried out using calibration curves generated from known concentrations (1–250 μg/mL) of mixed standard solutions. The results were expressed as μg/g dry weight. Validation parameters (limits of detection and quantification) have been established using standard deviation of response and slope in our previous analysis (Šola et al. 2023).

All chemicals and reagents used in the experiments were supplied by Sigma Aldrich GmbH (Taufkirchen, Germany), unless otherwise specified. Standards of flavonoids and phenolic acids were of HPLC grade and were purchased either from Sigma Aldrich GmbH (Taufkirchen, Germany) or from Extrasynthese (Genay, France).

### 2.3 Animals and Ethics Statement

The study utilized mice from a registered facility of the Department of Animal Physiology, Faculty of Science, University of Zagreb. Experimental animals were 3‐month‐old Swiss albino mice. In total, 30 animals of both sexes participated. The housing conditions were standard: 5 mice per cage, 12‐h light/12‐h dark cycle, temperature 22°C with controlled 60% humidity, with *ad libitum* access to food and water. Mice received a standard rodent diet (GLP standard diet, 4RF21, Mucedola, Settimo Milanese MI, Milan, Italy). Ethical approval for the experiments was obtained from the Ethics Committee of the Faculty of Science, University of Zagreb, Croatia (approval code: 251‐58‐10617‐14‐21; approval date: January 13, 2021). All animal care and maintenance followed guidelines in force in the Republic of Croatia (Law on Amendments to the Law on Animal Welfare, NN 37/13; Law on Animal Welfare, NN 102/2017; Regulation on the Protection of Animals Used for Scientific Purposes, NN 55/13) and according to the Guide for the Care and Use of Laboratory Animals, DHHS (NIH) Publ #86‐23 (National Research Council 2011).

### 2.4 Experimental Design

The animals were individually weighed and labeled before and during the experiment, and then assigned to groups with approximately similar body weights (25 ± 2 g). Each experimental group consisted of 10 animals, with an equal sex distribution (5 males and 5 females per group). Body weight was measured using a digital scale (ABS 220‐4, manufactured by Kern & Sohn, Balingen, Germany) at the beginning of the experiment, every 7 days during the experimental period, and on the day of euthanasia. Based on body weight, the quantity of each preparation administered during the experiment was calculated individually for each animal. The doses of extracts in all experimental groups corresponded to an intake of approximately 300 mg of dried plant extract/kg body weight/day, ensuring that all groups received an equal amount of plant material (extract) (Luo et al. 2024; Patil et al. 2024). The selected dose was based on previous animal studies demonstrating the biological activity and safety of broccoli‐derived extracts in mice, where commonly applied doses range from 50 to 800 mg/kg body weight (Jeong et al. 2025; Muthana et al. 2016). Furthermore, this dose was considered translationally relevant, as it corresponds to a feasible oral intake of concentrated broccoli‐derived bioactive compounds in humans through dietary supplements or lyophilized preparations. The altered content of bioactive compounds in the plants, resulting from different cultivation temperature conditions, was hypothesized to produce differing biological effects on mammalian physiology.

Throughout the 28‐day experimental period, animals received the respective treatments once daily by oral intragastric (*ig*) gavage using a gastric cannula. The experimental design included the following groups:

- Cont.—control group (0.3 mL of physiological saline).
- RT_extr._—group of animals treated with water extract from broccoli sprouts grown at 23°C (300 mg/kg of body weight per day).
- HT_extr._—group of animals treated with water extract from broccoli sprouts grown at 38°C (300 mg/kg of body weight per day).

At the end of the 28‐day experimental period, the animals were euthanized. Prior to euthanasia, they were appropriately anesthetized and provided with analgesia via intraperitoneal (*ip*) injection of a ketamine (Vetoquinol S.A., BP 189 Lure Cedex, France; active ingredient: Ketamine) – xylazine (Vetoquinol Biowet Sp., Gorzow, R. Poland; active ingredient: Xylazine) combination (25 mg/kg body weight). Following euthanasia, blood samples and cardiac tissue were collected for further analyses.

### 2.5 Blood Sampling and Analysis of Hematological Parameters

After adequate anesthesia, blood samples were collected by cardiac puncture. Blood was drawn into vacutainer tubes containing an anticoagulant (EDTA). The following hematological parameters were determined: total red blood cell count (RBC), hematocrit (Hct), hemoglobin (Hb), mean erythrocyte volume (MCV), mean content of hemoglobin in erythrocytes (MCH), mean hemoglobin concentration in erythrocytes (MCHC), erythrocyte size distribution (RDW), total white blood cell count (WBC), platelet count, and mean platelet volume (MPV). Hematological parameters were analyzed using a hematology analyzer Horiba ABX 169 (Micros, France).

### 2.6 Preparation of Cardiac Tissue Homogenate for Biomarker Measurement

A detailed description of the method for preparing cardiac tissue homogenates for the determination of metabolic, structural, anti‐inflammatory, and antioxidant biomarkers is provided in Ferara et al. (2025).

### 2.7 Quantitative Analysis of Atrial Natriuretic Peptide in Myocardial Tissue as an Indicator of Metabolic Status

The concentration of atrial natriuretic peptide (ANP) in myocardial tissue was quantified using a commercially available Mouse ANP ELISA kit (ELK Biotechnology Co. Ltd., Colorado, USA). Tissue homogenates were appropriately diluted and processed following the manufacturer's protocol. Absorbance was measured at 450 ± 10 nm using a CLARIOstar Plus microplate reader (BMG Labtech, Offenburg, Germany). ANP levels were calculated from a standard calibration curve generated with known concentrations of the analyte and expressed as pg/mg of protein. All samples were analyzed in duplicate, and dilution factors were included in the final calculations.

### 2.8 Assessment of Myocardial Structural Integrity via High‐Sensitivity Cardiac Troponin I (hs‐cTnI)

High‐sensitivity cardiac troponin I (hs‐cTnI) levels in myocardial tissue were quantified using a UniCel DxI 600 immunoassay system (Beckman Coulter, Indianapolis, IN, USA), based on chemiluminescent LOCI technology. The assay procedure was performed according to the manufacturer's instructions and in line with previously described methodology (Ferara et al. 2025). Briefly, appropriately prepared myocardial tissue homogenates were analyzed, and hs‐cTnI concentrations were calculated from the generated signal and expressed as μg/mg of protein. All samples were measured in duplicate, and dilution factors were accounted for in the final calculations.

### 2.9 Assessment of Inflammatory Markers in Myocardial Tissue: Matrix Metalloproteinases (MMP‐2, MMP‐9), Pentraxin 3 (PTX3), and Nitric Oxide (NO)

Inflammatory parameters in myocardial tissue were evaluated by quantifying nitric oxide (NO), pentraxin 3 (PTX3), and matrix metalloproteinases (MMP‐2 and MMP‐9) using spectrophotometric and immunoassay‐based approaches, with all samples analyzed in duplicate.

NO levels were determined using the Griess reaction with a commercial assay kit (Promega, Madison, WI, USA), as previously described in detail (Ferara et al. 2025). Briefly, absorbance was measured at 540 nm using a Tecan Infinite 200 microplate reader (Tecan, Hamburg, Germany). A sodium nitrite standard curve (0–100 μM) was used for quantification, and results were expressed as μM.

The concentrations of PTX3, MMP‐2, and MMP‐9 were measured in myocardial tissue homogenates using commercially available ELISA kits (ELK Biotechnology Co. Ltd., Colorado, USA) according to the manufacturer's instructions. Absorbance was recorded at 450 ± 10 nm using a CLARIOstar Plus microplate reader (BMG Labtech, Offenburg, Germany). Concentrations were calculated from standard calibration curves and expressed as ng/mg of protein.

### 2.10 Determination of Oxidative Stress Biomarkers in Myocardial Tissue by Measuring Malondialdehyde (MDA), Superoxide Dismutase (SOD) Activity, Catalase (CAT) Activity, and Total Glutathione (tGSH)

The obtained heart tissue supernatant was used for the determination of oxidative stress biomarkers, including lipid peroxidation (MDA), total glutathione (GSH), superoxide dismutase (SOD), and catalase (CAT) activity. Lipid peroxidation, GSH content, and CAT activity were determined spectrophotometrically following established protocols, as previously described (Oršolić et al. 2020). All parameters were normalized to protein content and expressed accordingly.

Lipid peroxidation was assessed by quantifying malondialdehyde (MDA) levels based on its reaction with thiobarbituric acid, forming a chromogenic complex measured at 532 nm using a Tecan Infinite 200 microplate reader (Hamburg, Germany). The MDA levels were determined using the molar absorption coefficient for the MDA‐TBA complex of 1.56 × 10^5^/M cm. The results were expressed in nmol/mg of protein.

Total glutathione (GSH) content was determined using Ellman's reagent (DTNB), with absorbance recorded at 440 nm using a Tecan Infinite 200 microplate reader (Hamburg, Germany). The concentration of total glutathione (tGSH) was calculated using the molar absorption coefficient ε(DTNB) = 8.22 M^−1^ cm^−1^ and expressed in mU/mg of protein.

Superoxide dismutase (SOD) activity was determined using a commercial Mouse T‐SOD ELISA kit (MyBioSource, San Diego, USA), according to the manufacturer's instructions, with absorbance measured at 440 nm using a Tecan Infinite 200 microplate reader (Hamburg, Germany), and the results were expressed in U/mg of protein.

Catalase (CAT) activity was determined by monitoring the decomposition rate of hydrogen peroxide at 240 nm over a defined time interval. Using a Tecan Infinite 200 microplate reader (Hamburg, Germany). Catalase activity was calculated using the extinction coefficient of H_2_O_2_ (ε = 39.4/mM cm), and the results were expressed in U/mg of protein.

### 2.11 Assessment of Antioxidant Capacity in Myocardial Tissue

#### 2.11.1 FRAP Method

The FRAP assay for tissue homogenates was performed according to the method described by Katalinic et al. (2005), adapted for animal tissues and modified from Benzie and Strain (1996). The FRAP reagent was prepared by mixing acetate buffer (300 mM, pH 3.6), TPTZ solution (10 mM in 40 mM HCl), and ferric chloride (20 mM) in a ratio of 10:1:1. The mixture was incubated for 40 min at 37°C. A volume of 50 μL of undiluted myocardial tissue homogenate was mixed with 1.5 mL of preheated FRAP reagent and 150 μL of distilled water. A reagent blank contained FRAP reagent and distilled water without sample, while the control consisted of distilled water and FRAP reagent without tissue homogenate. After 4 min of incubation, absorbance was measured at 593 nm using a Tecan Infinite 200 microplate reader (Tecan, Hamburg, Germany). A standard curve was generated using ferrous sulfate heptahydrate (0–1000 μM). The reducing power of iron in myocardial tissue homogenates was expressed as nmol Fe^2+^ per mg of total protein.

#### 2.11.2 ABTS Method

The ABTS assay for tissue homogenates was performed following the method described by Katalinic et al. (2005), adapted for animal tissues and modified from the assay by Re et al. (1999). The ABTS radical solution (ABTS•+) was prepared by oxidizing a 7 mM ABTS solution with a freshly prepared 140 mM potassium persulfate solution in a 1:1 ratio. On the day of analysis, the stock solution was diluted with phosphate buffer (pH 7.4) and incubated at 30°C until an absorbance of 0.700 (±0.020) was reached. A reagent blank consisted of 1 mL of phosphate buffer alone, while the control contained 1 mL of ABTS• + solution and 20 μL phosphate buffer without tissue homogenate. A Trolox standard solution was used for calibration. The results were expressed as mmol Trolox equivalents per mg of protein in the tissue homogenate.

### 2.12 Statistical Analysis

Descriptive statistical methods were used to characterize the distribution of the variables. Normality of data distribution was assessed using the Kolmogorov–Smirnov test. Data from broccoli microgreens (glucosinolates and polyphenolic compounds) followed a normal distribution and are presented as mean ± standard deviation. Differences between the two experimental groups (RT_extr._ and HT_extr_.) were analyzed using the Student's *t*‐test. Statistical significance was set at *p* ≤ 0.05. All other datasets did not follow a normal distribution and are presented as median with interquartile range. These include hematological parameters, metabolic parameters, structural parameters, inflammatory markers, oxidative stress markers, and antioxidant capacity. Differences among the experimental groups for non‐normally distributed data were analyzed using the Kruskal–Wallis test, followed by Dunn's post hoc test for multiple comparisons where appropriate. Statistical significance was set at *p* ≤ 0.05. Statistical analyses were performed using STATISTICA 14 software (StatSoft, Tulsa, OK, USA), and graphical representations were prepared using GraphPad Prism 7 (GraphPad Software 2020, La Jolla, CA, USA).

## 3 Results

### 3.1 Effect of High Growth Temperature on Glucosinolates and Different Groups of Polyphenolics in Broccoli Microgreens

As shown in Table 1, high temperature treatment (HT_extr_.) significantly affected the levels of total glucosinolates, total phenolics, and phenolic acids in broccoli sprouts. Total glucosinolates and phenolic acids decreased by 28% and 40%, respectively, whereas total phenolics increased by 30%. The effect of HT_extr_. on total flavonoids, hydroxycinnamic acids, and flavonols was not significant.

**TABLE 1:** Effect of high growth temperature on total glucosinolates and different groups of polyphenolics in broccoli microgreens.

	RT_extr._	HT_extr_.	ΔHT (%)
GLS (mg SinE/g dm)	22.70 ± 2.30	16.44 ± 2.41*	−28
TP (mg GAE/g dm)	8.10 ± 2.08	10.52 ± 2.36*	30
TF (mg QE/g dm)	21.78 ± 4.47	24.85 ± 8.98	14
TPA (mg CAE/g dm)	2.91 ± 0.54	1.73 ± 0.61*	−40
THCA (mg CAE/g dm)	5.52 ± 0.1	5.96 ± 0.79	8
TFlo (mg QE/g dm)	6.49 ± 0.31	7.06 ± 0.7	9

*Note:* Values represent average ± standard deviation of three biological and three technical replicas.

Abbreviations: CAE = caffeic acid equivalent, dm = dry mass, GAE = gallic acid equivalent, GLS = total glucosinolates, HT_extr_. = high temperature (*t* = 38°C day/33°C night), QE = quercetin equivalent, RT = room temperature (*t* = 23°C day/18°C night), SinE = sinigrin equivalent, TF = total flavonoids, TFlo = total flavonols, THCA = total hydroxycinnamic acids, TP = total polyphenolics, TPA = total phenolic acids.

* Statistically significant differences compared to the RT_extr_. group (Student's *t*‐test, *p* ≤ 0.05).

### 3.2 Effect of Broccoli Sprout Extracts on Hematological Parameters

The results of hematological parameters in the control and experimental groups after 28 days of daily intragastric administration of broccoli sprout extracts grown at 23°C and 38°C are presented in Table 2. Mean platelet volume (MPV) was significantly lower in both experimental groups (RT_extr_. and HT_extr_.) compared to the control group, while remaining hematological parameters did not show statistically significant differences between the groups.

**TABLE 2:** Hematological parameters after 28 days of administration of broccoli sprout extracts grown at different temperatures.

Blood parameter	Cont.	RT_extr_.	HT_extr_.
Red blood cells (×10^12^/L)	8.175 (6.263–9.2)	8.7 (6.3–9.5)	9.25 (6.5–9.4)
Hemoglobin (g/L)	122.5 (93.75–135.0)	130.0 (90.0–140.0)	135.0 (100.0–135.0)
Hematocrit (L/L)	0.4 (0.315–0.455)	0.44 (0.325–0.480)	0.45 (0.35–0.47)
MCV (fL)	55.4 (53.95–56.98)	55.8 (55.6–57.65)	57 (55.70–57.70)
MCH (pg)	14.85 (14.65–14.98)	14.70 (14.35–14.85)	14.60 (14.40–15.10)
MCHC (g/L)	267.0 (259.8–276.8)	264.0 (248.0–267.5)	262.0 (255.0–267.0)
RDW (%)	13.85 (13.43–14.18)	13.90 (13.45–14.15)	14.10 (13.50–14.40)
White blood cells (×10^9^/L)	0.8 (0.45–1.175)	0.8 (0.35–1.0)	1.1 (0.8–1.5)
Platelets (×10^9^/L)	129.0 (106.5–154.0)	133.0 (102.0–140.0)	94.0 (86.75–124.0)
MPV (fL)	7.150 (6.875–7.4)	6.8 (6.525–6.875)*	6.8 (6.6–6.9)*

*Note:* Results are shown as median with interquartile range for 10 animals per group. Statistical analysis was performed using the Kruskal–Wallis test followed by Dunn's post hoc test.

Abbreviations: cont. = control, HT_extr_. = extract of broccoli sprouts grown at 38°C, *ig* = intragastric, MCH = mean cell hemoglobin, MCHC = mean cell hemoglobin concentration, MCV = mean cell volume, MPV = mean platelet volume, RDW = red blood cell distribution width, RT_extr_. = extract of broccoli sprouts grown at 23°C.

* Statistically significant differences compared to the control group (*p* ≤ 0.05).

### 3.3 Metabolic Parameters—Atrial Natriuretic Peptid (ANP)

**FIGURE 1:**
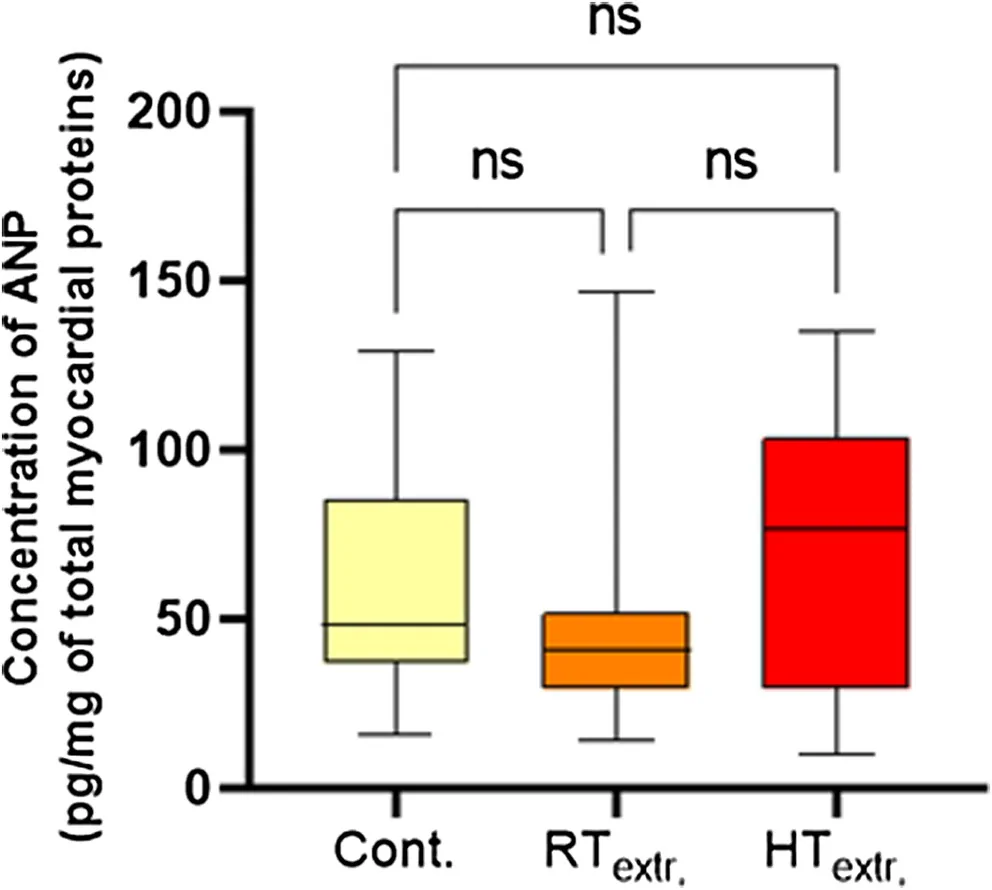
The concentration of atrial natriuretic peptide (ANP) in the control and experimental groups after 28 days of daily intragastric administration of broccoli sprout extracts grown at 23°C and 38°C is presented. Results are shown as median with interquartile range for 10 animals per group. Statistical analysis was performed using the Kruskal–Wallis test followed by Dunn's post hoc test. HT_extr._ = extract of broccoli sprouts grown at 38°C, *ig* = intragastric, cont. = control, RT_extr_. = extract of broccoli sprouts grown at 23°C.

No statistically significant differences in atrial natriuretic peptide (ANP) concentration in heart tissue homogenates were observed between the control and experimental groups (Figure 1).

### 3.4 Structural Parameters—High‐Sensitivity Cardiac Troponin I (Hs‐cTnI)

**FIGURE 2:**
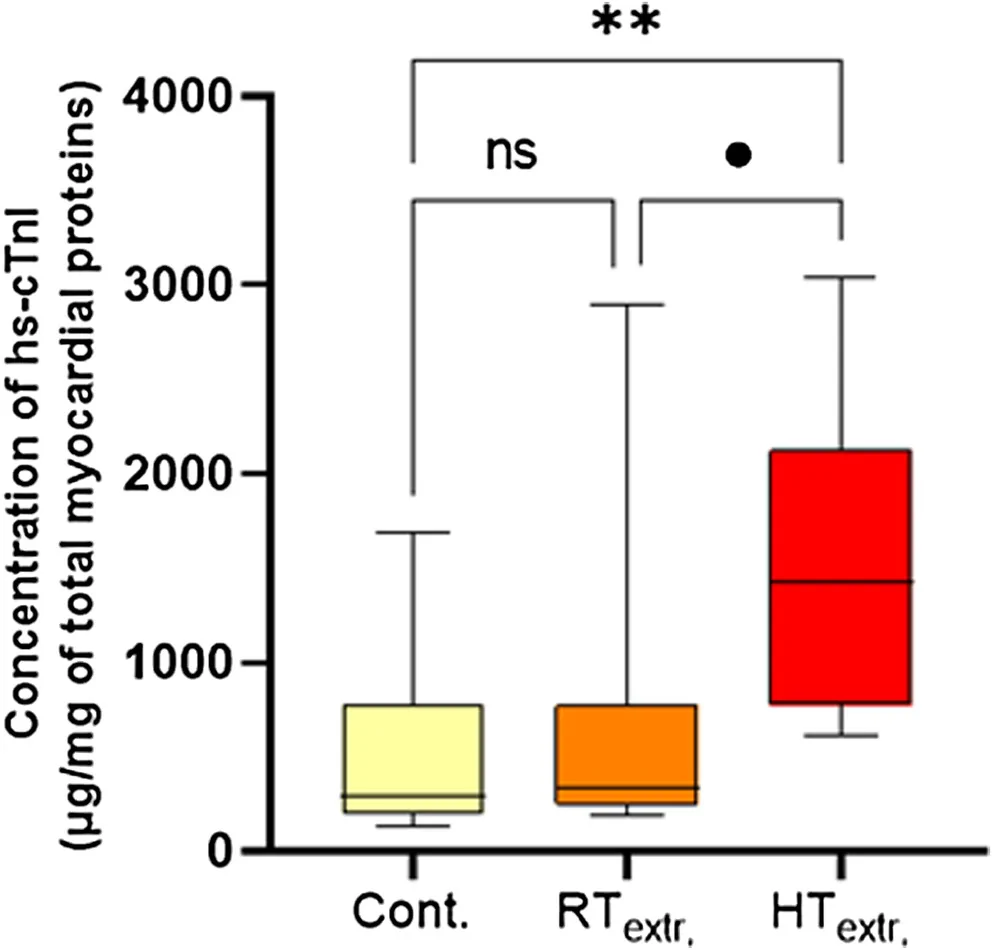
The concentration of high‐sensitivity cardiac troponin I (hs‐cTnI) in the control and experimental groups after 28 days of daily intragastric administration of broccoli sprout extracts grown at 23°C and 38°C. Results are shown as median with interquartile range for 10 animals per group. Statistical analysis was performed using the Kruskal–Wallis test followed by Dunn's post hoc test. *Statistically significant differences compared to the control group (**p ≤ 0.01). ^●^Statistically significant difference compared to the group treated with the extract of broccoli sprouts grown at 23°C (^●^ *p* ≤ 0.05). HT_extr._ = extract of broccoli sprouts grown at 38°C, *ig* = intragastric, cont. = control, RT_extr_. = extract of broccoli sprouts grown at 23°C.

Analysis of high‐sensitivity cardiac troponin (hs‐cTnI) concentrations in myocardial tissue homogenates revealed a significantly higher hs‐cTnI concentration in the group treated with the extract of broccoli sprout extracts grown 38°C aqueous extract of broccoli sprouts grown at 38°C (HT_extr_.) compared to both the control group (Cont.) (**p ≤ 0.01) and the group treated with the extract of broccoli sprouts grown at 23°C (RT_extr_.) (^●^ *p* ≤ 0.05) (Figure 2).

### 3.5 Inflammatory Parameters

**FIGURE 3:**
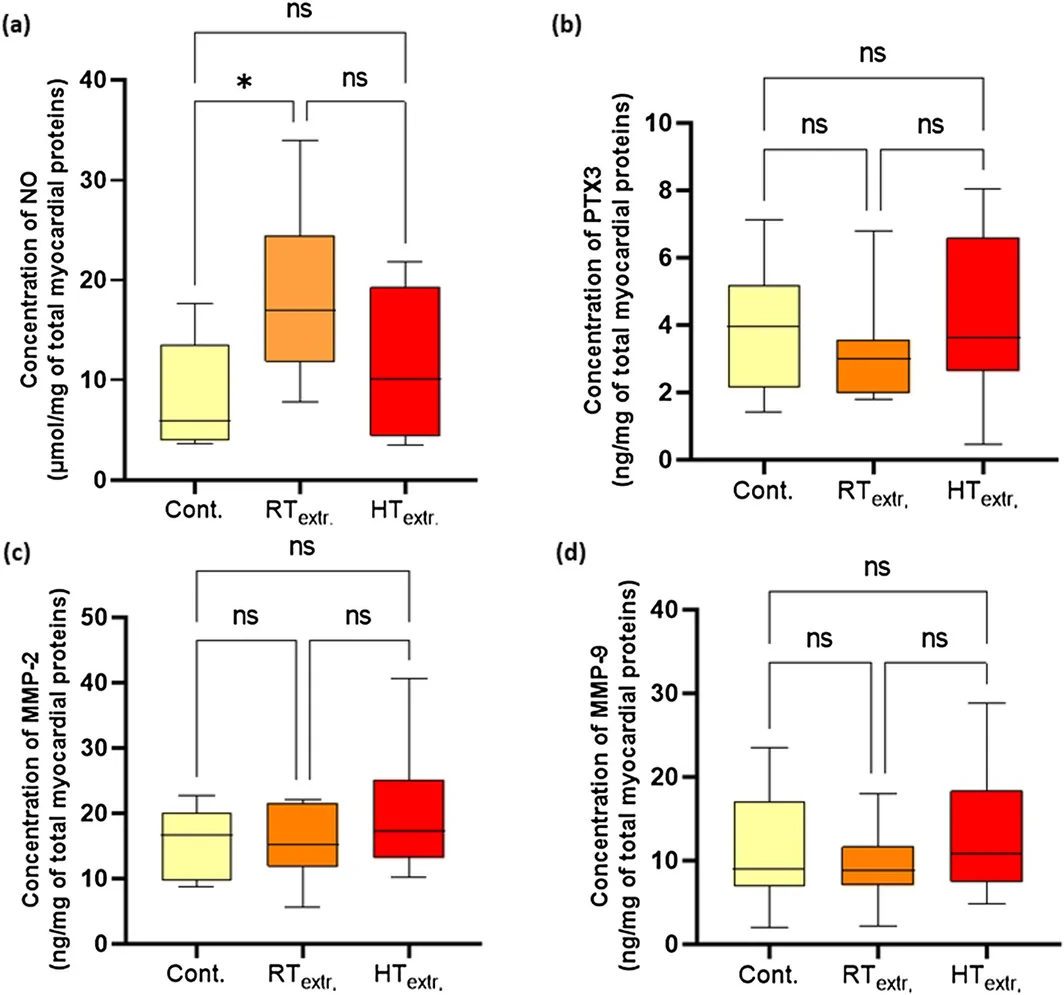
The concentrations of (a) nitric oxide (NO), (b) pentraxin 3 (PTX3), (c) matrix metalloproteinase‐2 (MMP‐2), and (d) matrix metalloproteinase‐9 (MMP‐9) in the control and experimental groups after 28 days of daily intragastric administration of broccoli sprout extracts grown at 23°C and 38°C. Results are shown as median with interquartile range for 10 animals per group. Statistical analysis was performed using the Kruskal–Wallis test followed by Dunn's post hoc test. *Statistically significant differences compared to the control group (*p ≤ 0.05). HT_extr._ = extract of broccoli sprouts grown at 38°C, *ig* = intragastric, cont. = control, RT_extr_. = extract of broccoli sprouts grown at 23°C.

Analysis of inflammatory biomarkers in heart tissue homogenates revealed a statistically significant increase in nitric oxide (NO) concentration in the RT_extr_. group compared to the control group (*p* ≤ 0.05) (Figure 3a). In contrast, no statistically significant differences were observed between the groups in the concentrations of pentraxin 3 (PTX3) or matrix metalloproteinases 2 and 9 (MMP‐2 and MMP‐9).

### 3.6 Oxidative Stress Biomarkers

**FIGURE 4:**
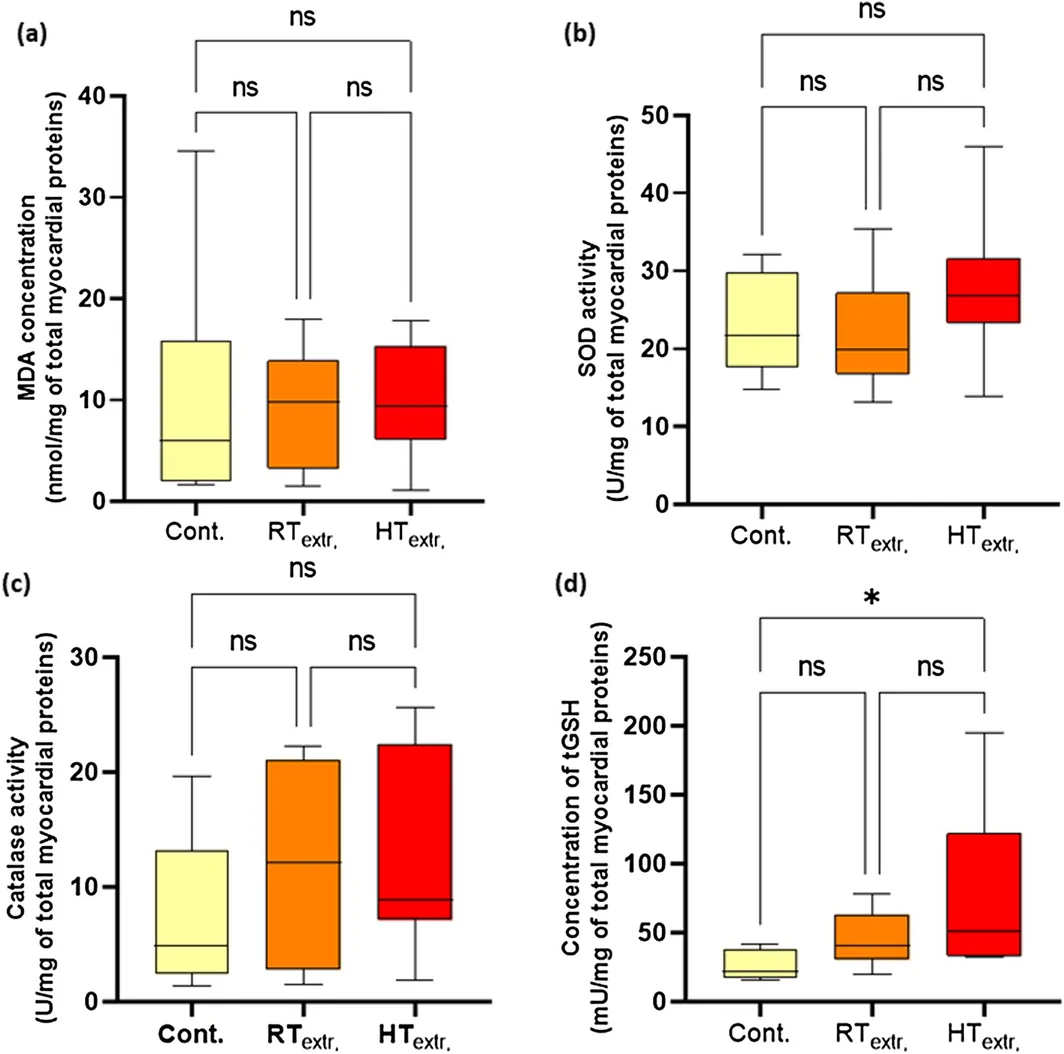
The concentrations of (a) malondialdehyde (MDA), (b) superoxide dismutase (SOD) activity, (c) catalase (CAT) activity, and (d) total glutathione (tGSH) in the control and experimental groups after 28 days of daily intragastric administration of broccoli sprout extracts grown at 23°C and 38°C. Results are shown as median with interquartile range for 10 animals per group. Statistical analysis was performed using the Kruskal–Wallis test followed by Dunn's post hoc test. *Statistically significant differences compared to the control group (*p ≤ 0.05). HT_extr._ = extract of broccoli sprouts grown at 38°C, *ig* = intragastric, cont. = control, RT_extr_. = extract of broccoli sprouts grown at 23°C.

The results of oxidative stress parameters are presented in Figure 4. Analysis of oxidative stress biomarkers revealed a statistically significant increase in total glutathione (tGSH) concentration in the HT_extr_. group compared to the control group (*p* ≤ 0.05) (Figure 4a). In contrast, no statistically significant differences were observed between the groups in malondialdehyde (MDA) concentration or in the activities of superoxide dismutase (SOD) and catalase (CAT).

### 3.7 Antioxidant Capacity

**FIGURE 5:**
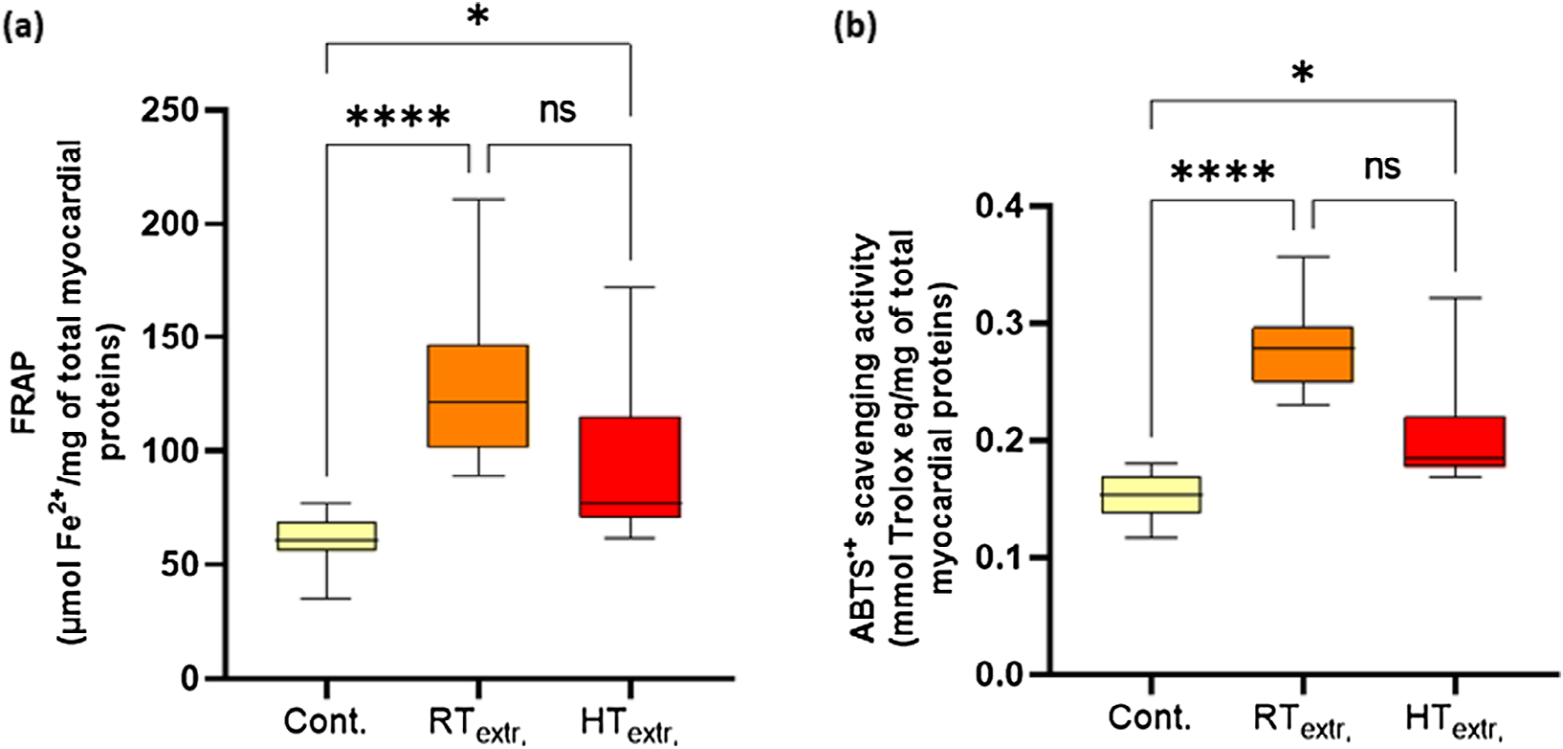
Antioxidant capacity of myocardial tissue measured using (a) the FRAP assay and (b) the ABTS assay in the control and experimental groups after 28 days of daily intragastric administration of broccoli sprout extracts grown at 23°C and 38°C. Results are shown as median with interquartile range for 10 animals per group. Statistical analysis was performed using the Kruskal–Wallis test followed by Dunn's post hoc test. *Statistically significant differences compared to the control group (*p ≤ 0.05; ****p ≤ 0.0001). HT_extr._ = extract of broccoli sprouts grown at 38°C, *ig* = intragastric, cont. = control, RT_extr_. = extract of broccoli sprouts grown at 23°C.

The analysis of antioxidant capacity, measured using the FRAP and ABTS assays, revealed a statistically significant increase in the antioxidant capacity of heart tissue in both the RT_extr_. group (*p* ≤ 0.0001) and the HT_extr_. group (*p* ≤ 0.05) compared to the control group (Figure 5a,b).

## 4 Discussion

Today's lifestyle and diet, characterized by low consumption of fruits and vegetables in many developed countries, contribute to metabolic stress and may compromise human health, highlighting the need for research into dietary strategies that support health maintenance and improvement. In the last decade, microgreens have gained increasing popularity both in culinary practice and among scientists and nutritionists, following numerous studies demonstrating that they are rich sources of phytochemicals beneficial to human health (Zhang et al. 2021). For this reason, microgreens, particularly those from the *Brassica* genus, are considered a promising new generation of functional foods due to their short production cycle and high concentrations of nutrients and bioactive compounds (Bhaswant et al. 2023; Belošević et al. 2024; Šola et al. 2020). However, despite their beneficial effects, some studies indicate that hydrolysis products of *Brassica*‐derived bioactive compounds, particularly isothiocyanates, may exhibit diverse biological activities and health effects (Di Gioia et al. 2020). Therefore, further research is required to better understand their mechanisms of action in the body and to evaluate the potential of broccoli sprouts as a functional food source rather than a so‐called “superfood”. This study investigated the in vivo effects of broccoli sprout extracts grown at 23°C and 38°C on hematological parameters and myocardial tissue in healthy Swiss mice. Metabolic, structural, inflammatory, and redox changes were assessed, as well as whether cultivation temperature alters the phytochemical composition of broccoli sprouts, potentially influencing blood and cardiac physiology in mammals after consumption.

In this study, hematological analysis revealed a statistically significant decrease in mean platelet volume (MPV) in both broccoli sprout extract‐treated groups compared to the control group (*p* ≤ 0.05) (Table 2). MPV is considered a marker of platelet size and is associated with platelet activation status, as larger platelets are generally more metabolically active and possess greater prothrombotic potential (Choi et al. 2016; Henshaw and Ogar 2018). However, since platelet counts remained unchanged, the isolated reduction in MPV should be interpreted cautiously and does not directly indicate altered platelet numbers. Within this context, the observed decrease in MPV may suggest a potential modulation of platelet reactivity. This is partially supported by previous studies reporting hematological and functional effects of broccoli‐derived compounds. For example, Al‐Qaraghli and Ayed (2023) observed improvements in hematological parameters, including platelet‐related indices, following broccoli seed supplementation in rats, while Van Steenwijk et al. (2023) reported a reduction in urinary 11‐dehydro‐thromboxane B_2_ after broccoli sprout consumption, indicating decreased in vivo platelet activation. Although the underlying mechanisms remain speculative, phytochemicals such as glucosinolates and polyphenols have been proposed to influence platelet function through multiple pathways, including modulation of NF‐κB signaling, reduction of thromboxane‐related activity, and effects on intracellular signaling cascades (Balykina et al. 2024; Fuentes et al. 2014; Ravishankar et al. 2018). Nevertheless, given the lack of direct functional platelet assays in this study, these findings should be interpreted as preliminary, and further targeted investigations are required to confirm their physiological relevance.

Growing evidence suggests that sulforaphane and broccoli extracts exert significant cardioprotective effects by attenuating oxidative stress and reducing inflammation. Pereyra et al. (2020) investigated the cardioprotective effects of orally administered sulforaphane‐enriched broccoli extract in a model of acute cardiac stress induced by intravenous isoproterenol (a sympathomimetic agent) in Sprague–Dawley rats. Their findings showed that the extract reduced sympathetic activity and enhanced parasympathetic tone over a 2‐week period, leading to normalization of heart rate and improvement in left ventricular function. Based on these results, the authors suggested that sulforaphane‐enriched broccoli extract may contribute to the regulation of autonomic cardiac function and provide protection against acute cardiac stress. In a rabbit model of chronic heart failure induced by aortic constriction, sulforaphane, a bioactive compound present in broccoli extract, was associated with reduced levels of atrial natriuretic peptide (ANP), brain natriuretic peptide (BNP), malondialdehyde, interleukin‐6, and collagen types I and III, as well as increased superoxide dismutase (SOD) activity over a 12‐week period (Ma et al. 2018). These changes were accompanied by improvements in cardiac function and remodeling, potentially mediated through the attenuation of oxidative stress and inflammation. Additionally, the cardioprotective effects of sulforaphane have been reported in animal models of myocardial infarction. In these studies, sulforaphane was associated with reduced cardiac fibrosis, decreased levels of pro‐inflammatory cytokines such as interleukin‐1, interleukin‐6, and tumor necrosis factor‐α (TNF‐α), reduced reactive oxygen species (ROS) production, and increased levels of matrix metalloproteinases, SOD activity, and nitric oxide (NO) (Fernandes et al. 2016; Kee et al. 2015; Silva‐Palacios et al. 2019).

In contrast to these findings, analysis of atrial natriuretic peptide (ANP) concentration in the current study, a biomarker of hemodynamic load, revealed no statistically significant differences between the experimental and control groups (Figure 1). In our previous study, however, intragastric administration of the pure glucosinolate sinigrin resulted in a significant reduction in ANP expression in cardiac tissue homogenates, although this effect was observed only in female mice (Ferara et al. 2025). One possible explanation for the absence of a significant effect following administration of broccoli sprout extracts may lie in their complex and heterogeneous composition. In addition to sinigrin, the extracts contain other glucosinolates as well as polyphenols, which may exert multiple, potentially interacting or even counteracting biological effects. Alternatively, the concentration of bioactive compounds may have been insufficient to elicit a measurable response under the applied conditions. Moreover, several methodological differences should be considered when comparing these findings with previous studies. Earlier research often employed longer treatment durations, higher or standardized doses of bioactive compounds such as sulforaphane, and pathological models characterized by pre‐existing cardiac dysfunction. For instance, Xu et al. (2016) used doses standardized to sulforaphane equivalents of up to 1 mg/kg, while lower doses (0.5 mg/kg) also produced beneficial effects, albeit to a lesser extent. In the present study, the experimental conditions involved a 28‐day treatment period and administration of 300 mg of dry broccoli sprout extract per kg of body weight, without standardization to a specific bioactive compound. Based on total glucosinolate content expressed as sinigrin equivalents (room temperature: 22.70 ± 2.30 mg SinE/g dry weight; high temperature: 16.44 ± 2.41 mg SinE/g dry weight), the estimated daily intake corresponded to 6.81 mg SinE/kg body weight for the RT extract and 4.93 mg SinE/kg body weight for the HT extract. These values are comparable but still lower than the 10 mg/kg body weight dose of sinigrin previously shown to induce a significant reduction in ANP levels. Taken together, these differences in dose, duration, and extract standardization may have contributed to the absence of a detectable effect on ANP expression. It is therefore plausible that the administered extracts did not provide a sufficiently high concentration of specific bioactive constituents, such as sinigrin or its derivatives, to elicit measurable changes under the conditions of this study. Furthermore, the use of healthy animals, as opposed to disease models, may additionally limit the sensitivity for detecting changes in cardiac biomarkers.

Further, a statistically significant increase (*p* ≤ 0.05) in myocardial hs‐cTnI levels was observed in the group treated with broccoli sprout extract grown at high temperature (HT_extr_.) compared to both the room temperature extract group (RT_extr_.) and the control group (*p* ≤ 0.01) (Figure 2). These findings are not in line with previous studies reporting cardioprotective effects of broccoli‐derived compounds, particularly glucosinolates and polyphenols. Several studies have demonstrated protective effects of sulforaphane and broccoli sprout extracts in models of cardiac injury, including reduced inflammatory signaling, oxidative stress, and markers of myocardial damage such as cTnI, CK‐MB, LDH, and AST, as well as improved histological and functional outcomes (Akhlaghi and Bandy 2010; Hadi et al. 2023; Li et al. 2022; Sergazy et al. 2020; Singh et al. 2015). In contrast to these reports, the present results suggest a potential increase in myocardial stress associated with the HT_extr_., as no such effect was observed in the RT_extr_. group. This difference may be linked to temperature‐induced alterations in phytochemical composition. Specifically, compared to the RT_extr_., the HT_extr._ contained 28% lower total glucosinolates and 40% lower phenolic acids, while total phenolic content increased by 30% (Table 1). However, this increase in total phenolics does not necessarily correspond to enhanced cardioprotective capacity, as bioactivity depends strongly on the specific phenolic profile and synergistic interactions with glucosinolates. Importantly, the observed increase in myocardial hs‐cTnI should be interpreted with caution. Although hs‐cTnI is widely used as a sensitive marker of cardiomyocyte injury in circulation, its interpretation in tissue homogenates is less straightforward and does not allow definitive conclusions regarding irreversible myocardial damage. Therefore, the elevated hs‐cTnI levels may reflect altered myocardial stress responses or changes in cellular turnover rather than direct tissue injury. Overall, these findings suggest that cultivation temperature may significantly influence the phytochemical composition of broccoli sprout extracts and, consequently, modulate cardiac‐related molecular responses. However, the biological significance and mechanistic basis of the observed changes in hs‐cTnI remain to be fully elucidated.

Additionally, inflammatory parameters, including MMP‐2, MMP‐9, and PTX3, did not differ significantly among the experimental groups, whereas myocardial NO concentration was significantly increased in the HT_extr_. group (Figure 3). Nitric oxide (NO) is a bioactive gaseous molecule with well‐established vasodilatory, anti‐inflammatory, and antioxidant properties. Its biological effects are primarily mediated through activation of the cGMP–protein kinase G signaling pathway, resulting in reduced intracellular calcium influx and smooth muscle relaxation (Aekthammarat et al. 2020; Iqbal et al. 2023). NO is synthesized by three nitric oxide synthase isoforms (nNOS, iNOS, and eNOS), which catalyze the conversion of L‐arginine to NO and L‐citrulline in an NADPH‐dependent reaction (Frąk et al. 2022). Under physiological conditions, endothelial NOS (eNOS) is the principal regulator of vascular NO homeostasis. However, this finely balanced system is highly susceptible to disruption under oxidative stress, which may impair eNOS function and contribute to endothelial dysfunction. In such conditions, dysregulation of enzymatic systems, including NADPH oxidases, xanthine oxidase, and uncoupled NOS, further amplifies reactive oxygen species (ROS) production, thereby promoting vascular dysfunction, inflammation, and altered vascular tone (Mourouzis et al. 2021). Within this redox‐sensitive context, glucosinolates are generally associated with indirect antioxidant effects mediated through Nrf2 activation and upregulation of endogenous antioxidant defenses, rather than direct stimulation of NO synthesis (Xu et al. 2016). Interestingly, activation of the Nrf2 pathway under oxidative conditions has been shown to increase NO bioavailability while simultaneously reducing eNOS expression, which is considered an adaptive response aimed at preserving endothelial function and preventing eNOS uncoupling (Heiss et al. 2009; Shehatou and Suddek 2016). This involves improved tetrahydrobiopterin (BH4) availability and attenuation of oxidative stress, thereby limiting superoxide generation from eNOS. In this framework, glucosinolates may indirectly support NO homeostasis by stabilizing redox balance and maintaining eNOS functionality, whereas polyphenols are more directly implicated in NO upregulation through induction of eNOS/iNOS expression and activation of signaling pathways such as SIRT1 (Gál et al. 2023; Iqbal et al. 2023). Against this mechanistic background, the significantly higher myocardial NO concentration observed in the HT_extr_. group may be associated with its higher polyphenolic content compared with RT_extr_. However, this interpretation is complicated by the concurrent reduction in total glucosinolate content in the same group. Although glucosinolates are not primary inducers of NO synthesis, their role in maintaining eNOS stability and cellular redox equilibrium may still be relevant for preserving NO bioavailability in myocardial tissue. Consequently, the observed NO increase likely reflects a complex interaction between phytochemical classes rather than the isolated effect of a single compound group, particularly in the myocardium where NO signaling is highly sensitive to metabolic and redox fluctuations.

Despite the observed modulation of NO levels, treatment with aqueous broccoli sprout extracts did not significantly affect myocardial MMP‐2 or MMP‐9 concentrations compared with the control group (Figure 3c,d). Comparisons with previous studies are limited because most available research has focused on isolated glucosinolates or purified polyphenolic compounds rather than crude broccoli sprout extracts. Nevertheless, Xu et al. (2016) demonstrated that high doses of broccoli sprout extract attenuated myocardial fibrosis in a mouse model of type II diabetes, as evidenced by reduced collagen accumulation and decreased connective tissue growth factor (CTGF) expression, indirectly suggesting a potential role in modulating MMP‐related cardiac remodeling. In support of this concept, Yun and Kim (2024) reported that broccoli sprout extract suppressed MMP‐1 expression and inflammatory mediators, including cyclooxygenase‐2 (COX‐2) and interleukin‐6 (IL‐6), in keratinocyte cultures through selective inhibition of the MAPK signaling pathway via direct interaction with p38α. However, unlike these studies, the present investigation was conducted under physiological conditions without experimentally induced inflammation or fibrosis, which may explain the absence of detectable changes in myocardial MMP concentrations.

Consistent with these findings, analysis of pentraxin 3 (PTX3) concentrations also revealed no statistically significant differences between broccoli sprout‐treated groups and the control group (Figure 3b). Unlike C‐reactive protein, PTX3 is produced locally at sites of inflammation and reflects acute tissue injury with rapid kinetics (Chodkowski et al. 2018; Latini et al. 2004; Ristagno et al. 2019). Therefore, the absence of significant changes in PTX3 concentrations suggests that subchronic administration of broccoli sprout extracts did not induce acute myocardial injury or inflammatory activation. Considering that PTX3 concentrations typically peak within 6–8 h after acute myocardial damage (Ristagno et al. 2019), substantial alterations were not expected under the present subchronic experimental conditions. To the best of our knowledge, this is the first study investigating the effects of broccoli sprout extracts on myocardial PTX3 concentrations. Although PTX3 is widely recognized as an independent prognostic marker of adverse cardiovascular outcomes and is associated with left ventricular dysfunction and metabolic disorders (Ding et al. 2022; Matsubara et al. 2011; Ristagno et al. 2019), accumulating evidence suggests a more complex biological role. In addition to serving as a biomarker of tissue injury, PTX3 may also exert cardioprotective effects, as PTX3 deficiency has been associated with enhanced vascular inflammation and accelerated atherosclerotic progression (Chodkowski et al. 2018). Therefore, maintenance of stable PTX3 concentrations in the present study may further support the absence of significant inflammatory or pathological remodeling processes within the myocardium following broccoli sprout extract treatment.

In parallel with these inflammatory findings, oxidative stress parameters showed no significant differences among the experimental groups, with the exception of total glutathione concentration (Figure 4). Myocardial MDA levels remained unchanged following treatment with broccoli sprout extracts cultivated at different ambient temperatures, suggesting that lipid peroxidation was not significantly affected under physiological conditions. Consistently, catalase and SOD activities were also unaltered. Since these enzymes function sequentially in reactive oxygen species detoxification and redox homeostasis (Skoryk and Horila 2023; Khan and Younus 2025), the absence of changes suggests that the treatment neither disrupted basal oxidative balance nor triggered compensatory activation of primary enzymatic antioxidant defenses. Taken together, these observations indicate that the antioxidant effects of broccoli‐derived phytochemicals, particularly with respect to MDA and enzymatic antioxidants, are more evident under conditions of oxidative challenge or tissue injury, where they contribute to restoration of impaired redox homeostasis rather than modulation of physiological baseline status.

In contrast to these parameters, a significantly higher myocardial total glutathione (tGSH) concentration was observed in the HT_extr_. group compared with the control group (Figure 4d). Glutathione represents the most abundant intracellular non‐enzymatic antioxidant in the myocardium and plays a central role in maintaining cardiac redox balance (Labarrere and Kassab 2022). Total glutathione reflects the combined pool of reduced and oxidized forms and thus provides an integrated measure of glutathione system capacity (Townsend et al. 2003). Elevated tGSH levels are generally associated with enhanced antioxidant buffering capacity and improved cellular resilience to oxidative stress (Tan et al. 2023), suggesting a potential modulation of endogenous defense mechanisms. Nevertheless, this finding should be interpreted cautiously. Myocardial glutathione concentrations are physiologically lower than in many other tissues due to reduced γ‐glutamyl transpeptidase activity, meaning that even modest absolute changes may be biologically relevant (Salyha 2020). Moreover, increased tGSH may reflect either enhanced antioxidant capacity or a compensatory response to subtle redox perturbations (Emeka et al. 2021). Importantly, total glutathione measurement does not distinguish between reduced (GSH) and oxidized (GSSG) forms, whereas the GSH/GSSG ratio provides a more precise indicator of intracellular redox status (Dulce Bagatini 2020; Zanginyan et al. 2019). Consequently, without assessment of this ratio, it remains difficult to determine whether the observed increase reflects true enhancement of antioxidant defense or adaptive metabolic adjustment.

Antioxidant capacity represents the ability of antioxidant molecules to inhibit oxidative processes through free radical scavenging, metal chelation, or inhibition of pro‐oxidant enzymes (Ghiselli et al. 2000). In this study, FRAP and ABTS assays revealed a statistically significant increase in myocardial antioxidant capacity in the experimental groups RT_extr._ (*p* ≤ 0.0001) and HT_extr_. (*p* ≤ 0.05) compared to the control group (Figure 5a,b). It is interesting to compare these findings with our previous study, which did not observe an increase in myocardial antioxidant capacity following subchronic administration of pure sinigrin (Ferara et al. 2025). One possible explanation is that broccoli sprout extracts represent complex mixtures of biologically active phytochemicals, which may exert a broader and more pronounced effect on total antioxidant capacity compared to a single glucosinolate such as sinigrin. In addition, the presence of polyphenols likely contributes to this difference. Although both glucosinolates and polyphenols are recognized as antioxidants, polyphenols may exert their effects more directly. They can act as direct free radical scavengers by donating electrons or hydrogen atoms through their aromatic structures and can also chelate transition metal ions such as Fe^2+^/Fe^3+^ and Cu^2+^, thereby limiting hydroxyl radical formation via the Fenton reaction. In addition, polyphenols may exert indirect antioxidant effects by modulating the activity of enzymes such as catalase, superoxide dismutase (SOD), and glutathione peroxidase, as well as by inhibiting pro‐oxidant enzymes including xanthine oxidase and NADPH oxidase (Iqbal et al. 2023). In contrast, glucosinolates exert their antioxidant effects primarily through activation of the Nrf2/ARE signaling pathway, which regulates the expression of multiple antioxidant and phase II detoxification enzymes, including SOD, catalase, heme oxygenase‐1, glutathione peroxidase, thioredoxin, NAD(P)H:quinone oxidoreductase 1 (NQO1), and glutathione S‐transferase (Corssac et al. 2018). In simplified terms, polyphenols can be considered direct antioxidants, whereas glucosinolates mainly act as indirect, enzyme‐mediated antioxidants.

Given that FRAP and ABTS assays primarily reflect electron or hydrogen‐donating capacity, it is expected that polyphenols would exhibit higher apparent antioxidant activity than glucosinolates. These assays are not designed to capture indirect, signaling‐mediated antioxidant effects such as those mediated by the Nrf2 pathway. Indeed, in vitro studies have shown that phenolic compounds such as myricetin and quercetin derivatives exhibit substantially stronger antioxidant activity in FRAP, ABTS, and DPPH assays compared to glucosinolate‐derived isothiocyanates (Fusari et al. 2020). However, these findings cannot be directly extrapolated to in vivo systems. Importantly, in the present study, antioxidant capacity was assessed in myocardial tissue rather than in the plant extracts. Both glucosinolates and polyphenols are characterized by limited bioavailability and undergo extensive metabolism and biotransformation prior to reaching target tissues (Di Lorenzo et al. 2021; Hossain et al. 2025; Prieto et al. 2019). Furthermore, glucosinolates are biologically inactive until converted by myrosinase into isothiocyanates, meaning that their bioactivity depends on enzymatic conversion in the gastrointestinal tract or tissues (Barba et al. 2016). Distribution studies using radiolabeled isothiocyanates indicate that the highest accumulation occurs in the intestines, liver, kidneys, urinary bladder, lungs, and spleen, while the heart shows comparatively lower uptake (Prieto et al. 2019). In contrast, polyphenols may accumulate in peripheral tissues after absorption. Reported distribution patterns suggest the following order: kidneys > liver > lungs > heart > muscle (Matsui 2022). Moreover, increased tissue accumulation of dietary polyphenols has been demonstrated, including higher concentrations in liver and myocardium compared to plasma (Wallace et al. 2006), as well as elevated flavonoid and phenolic levels in cardiac tissue following dietary supplementation (Udomkasemsab et al. 2018). Overall, despite their limited bioavailability, polyphenols may accumulate in the myocardium and thereby contribute to local antioxidant effects (Balta et al. 2020, 2023). In conclusion, the observed increase in myocardial antioxidant capacity following broccoli sprout extract administration is most likely the result of a synergistic interaction between glucosinolates and polyphenols.

## 5 Conclusions

In conclusion, the findings of this in vivo study suggest that aqueous broccoli sprout extracts may exert a potential antiplatelet effect, as indicated by the reduction in mean platelet volume (MPV), and may enhance overall myocardial antioxidant capacity, likely through the combined action of polyphenols and glucosinolates. However, the results also indicate that cultivation at elevated temperature (38°C; HT) substantially alters the phytochemical profile of the plant, particularly by reducing total glucosinolates and phenolic acids, which may contribute to differential physiological responses in myocardial tissue. While the room temperature extract (RT_extr_.) was associated with increased myocardial nitric oxide (NO) levels, the HT_extr_. was associated with a statistically significant elevation in myocardial hs‐cTnI. Although this finding may suggest a potential adverse or stress‐related cardiac response linked to the altered phytochemical composition of the HT_extr_., it cannot be interpreted as definitive evidence of myocardial injury in the absence of corresponding serum troponin measurements and additional functional markers. Furthermore, given the lack of significant changes in ANP levels, the limited bioavailability of these phytochemicals, and the inherent limitations of extrapolating in vitro antioxidant assays to in vivo conditions, these results should be interpreted with caution. Further studies, particularly using pathological models and detailed pharmacokinetic analyses, are warranted to clarify the underlying mechanisms and to determine whether temperature‐induced phytochemical changes in microgreens may contribute to cardioprotective or potentially adverse cardiac effects.

## 6 Study Limitations and Future Research Directions

Despite the promising findings, several limitations should be considered. The antiplatelet effect was inferred from the reduction in mean platelet volume (MPV) without direct assessment of platelet function or aggregation, while the increase in myocardial hs‐cTnI should be interpreted cautiously because serum troponin was not measured. In addition, the study was conducted in healthy mice over a relatively short period, limiting the extrapolation of the findings to pathological cardiovascular conditions. Although both sexes were equally represented in the experimental groups, sex‐specific analyses were not performed; therefore, potential sex‐related differences in the response to broccoli sprout extracts cannot be excluded. Furthermore, the bioavailability of the bioactive compounds and their tissue distribution were not determined, and detailed molecular, histological, and functional cardiac assessments were not performed.

Nevertheless, the present findings demonstrate that cultivation temperature can substantially modify the phytochemical composition of broccoli sprouts, resulting in distinct physiological responses in vivo. These observations highlight the importance of optimizing cultivation conditions while emphasizing that the biological effects of temperature‐modified functional foods should be confirmed by comprehensive in vivo evaluation rather than by in vitro antioxidant capacity alone. Future studies should validate these findings in pathological cardiovascular models, investigate the pharmacokinetics and bioavailability of key phytochemicals, evaluate potential sex‐specific responses, and incorporate functional and mechanistic analyses to better define the safety and cardioprotective potential of broccoli sprout extracts.

## Author Contributions

**Nikola Ferara:** investigation, formal analysis, data curation, writing – review and editing. **Irena Landeka Jurčević:** writing – review and editing, investigation, data curation, conceptualization, methodology, validation. **Vedran Balta:** writing – original draft, supervision, software, validation, conceptualization, investigation, methodology, writing – review and editing, visualization, formal analysis, data curation. **Anđelo Beletić:** investigation, formal analysis, data curation, writing – review and editing. **Ivana Šola:** project administration, resources, funding acquisition, software, validation, investigation, conceptualization, methodology, writing – review and editing, visualization, formal analysis, data curation. **Domagoj Đikić:** conceptualization, investigation, data curation, validation, writing – review and editing, methodology. **Dyana Odeh:** writing – review and editing, investigation, data curation, formal analysis.

## Funding

This work was supported by the Croatian Science Foundation research project “Indirect effect of global warming on mammals' physiological parameters via high temperature‐stressed plant diet (TEMPHYS),” grant number IP‐2020‐02‐7585 (I.Š.).

## Ethics Statement

This study was conducted according to the guidelines of the Declaration of Helsinki and approved by the Ethical Committee of the Faculty of Science, University of Zagreb, Croatia (approval code: 251‐58‐10617‐14‐21; date of approval: 13 January 2021).

## Consent

The authors have nothing to report.

## Conflicts of Interest

The authors declare no conflicts of interest.

## Data Availability

The authors confirm that the data supporting the findings of this study are available within the article.
